# Supplementary material for: Selective enrichment of plasma cell-free messenger RNA in cancer-associated extracellular vesicles
Source: Commun Biol. 2023 Aug 29;6:885. doi: 10.1038/s42003-023-05232-z (PMC10465482; doi:10.1038/s42003-023-05232-z)
Supplement: Supplementary file 2 — Supplementary Information [file 42003_2023_5232_MOESM2_ESM.pdf]

Supplementary Information for

Selective enrichment of plasma cell-free messenger RNA in cancer-associated extracellular vesicles

Hyun Ji Kim[1,2], Matthew J. Rames[1,2], Florian Goncalves[1,2], C. Ward Kirschbaum[1], Breeshey Roskams-Hieter[1], Elias Spiliotopoulos[1], Josephine Briand[1], Aaron Doe[1], Joseph Estabrook[1,3], Josiah T. Wagner[1,4], Emek Demir[1,3,5], Gordon Mills[6], Thuy T. M. Ngo[1,2,5,6*]

[1]Cancer Early Detection Advanced Research Center (CEDAR), Knight Cancer Institute, Oregon Health and Science University, Portland, OR, USA.
[2]Department of Biomedical Engineering, Oregon Health and Science University, Portland, OR, USA.
[3]Computational Biology Program, Oregon Health and Science University, Portland, OR, USA.
[4]Molecular Genomics Laboratory, Providence Health and Services, Portland, OR, USA.
[5]Department of Molecular and Medical Genetics, Oregon Health and Science University, Portland, OR, USA.
[6]Division of Oncological Sciences, Knight Cancer Institute, Oregon Health and Science University, Portland, OR, USA.

*Thuy T. M. Ngo
Email: ngth@ohsu.edu

This PDF file includes:

Supplementary Figures 1 to 12

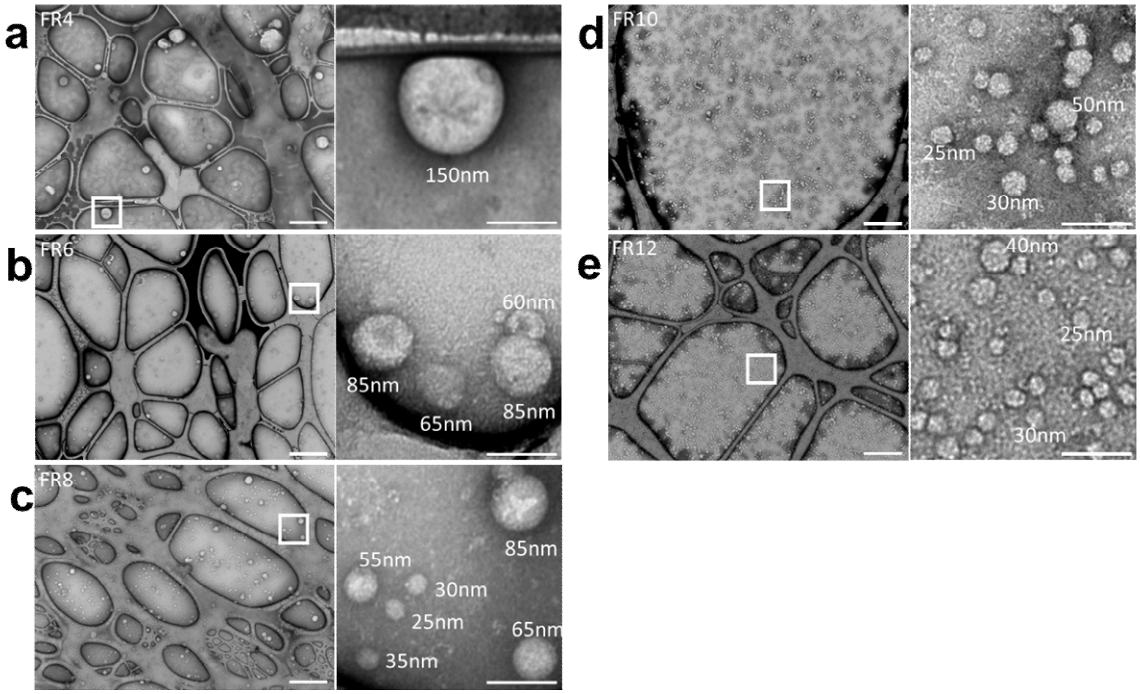

Supplementary Fig. 1. Transmission electron microscopy (TEM) imaging analysis of particles collected from size fractionation of human plasma. Representative TEM images are shown for **(a)** FR4, **(b)** FR6, **(c)** FR8, **(d)** FR10, and **(e)** FR12 eluted from the size fractionation column. Inset shows a magnified portion of the image. Scale bars are 500 nm and 100 nm for low-magnification and particle zoom-ins respectively.

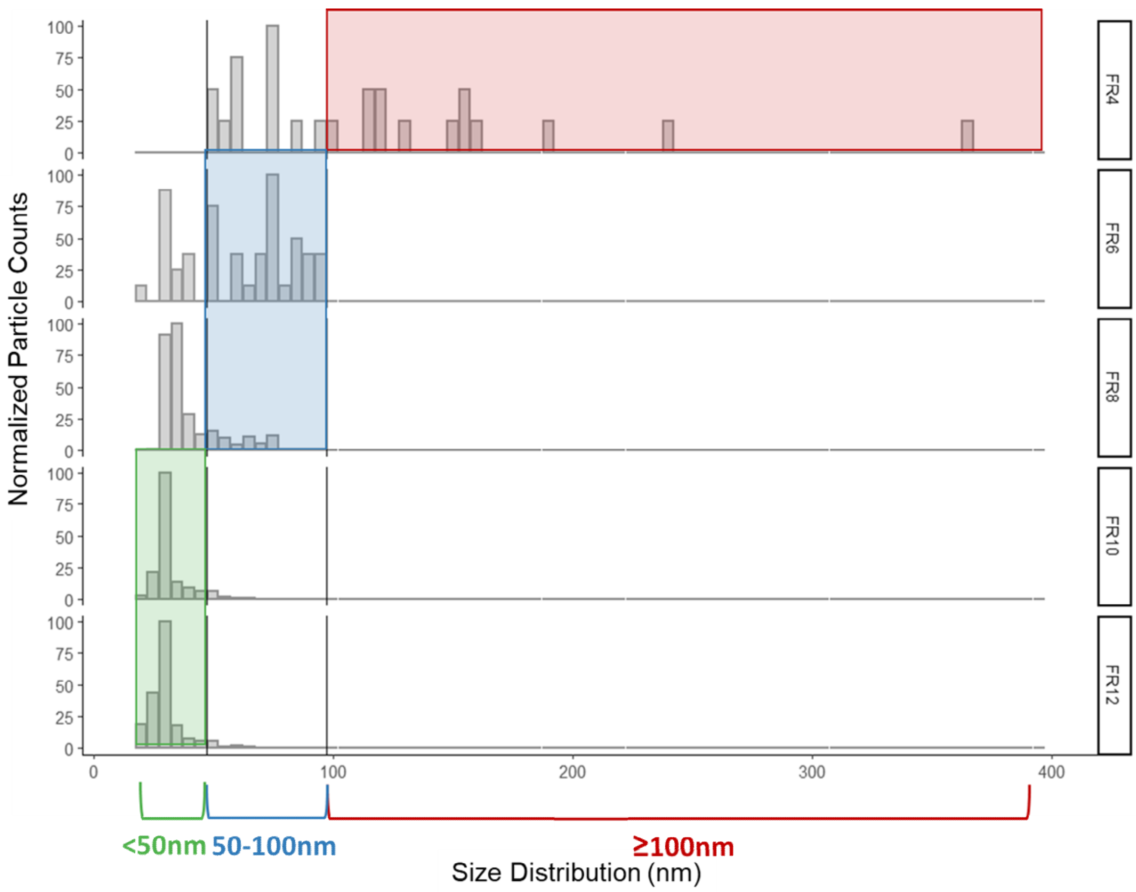

Supplementary Fig. 2. Size distribution quantification by transmission electron microscopy. Histograms of particle size distribution for normalized counts measured by TEM are shown for each fraction. Each bin covers a range of 5 nm increments for particle diameters. Areas with corresponding size ranges are shown: ≥ 100 nm (red), ≥ 50 nm & < 100 nm (blue), and < 50 nm (green).

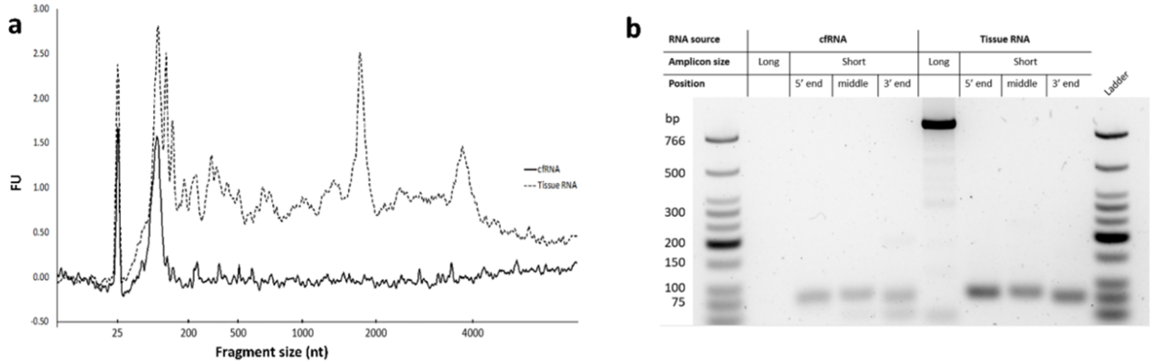

Supplementary Fig. 3. Plasma cell-free RNA length distribution. (a) Agilent Bioanalyzer analysis of size distribution of total cfRNA extracted from human plasma and 1 ng/µL of tissue RNA. Most cfRNA fragments fell between the 25 nt lower marker and 200 nt, while tissue RNA showed a much larger fragment size distribution. **(b)** Agarose gel electrophoresis of gene-specific PCR amplificons from cfRNA and tissue RNA. Four primer sets were designed to target different regions and amplicon lengths for the gene ALB. One "Long" primer pair was designed to amplify an 898 bp fragment of ALB, and three "Short" primer pairs were designed to amplify approximately 80 bp fragments along the 5', middle section, and 3' end of the gene. Upon RT-PCR, tissue RNA amplified fragments across all short and long primer pairs, while cfRNA only showed amplification for short amplicon fragments.

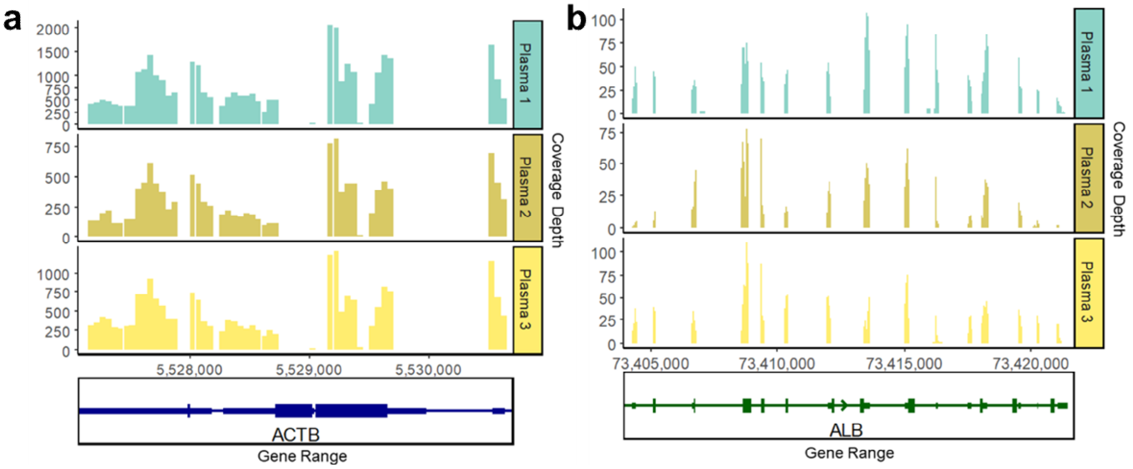

Supplementary Fig. 4. Coverage plot of transcriptomic data set. Coverage track was generated on **(a)** ACTB and **(b)** ALB genes from cell-free RNA sequencing data using the bedtool utilities and visualized using the ggcoverage package in R.

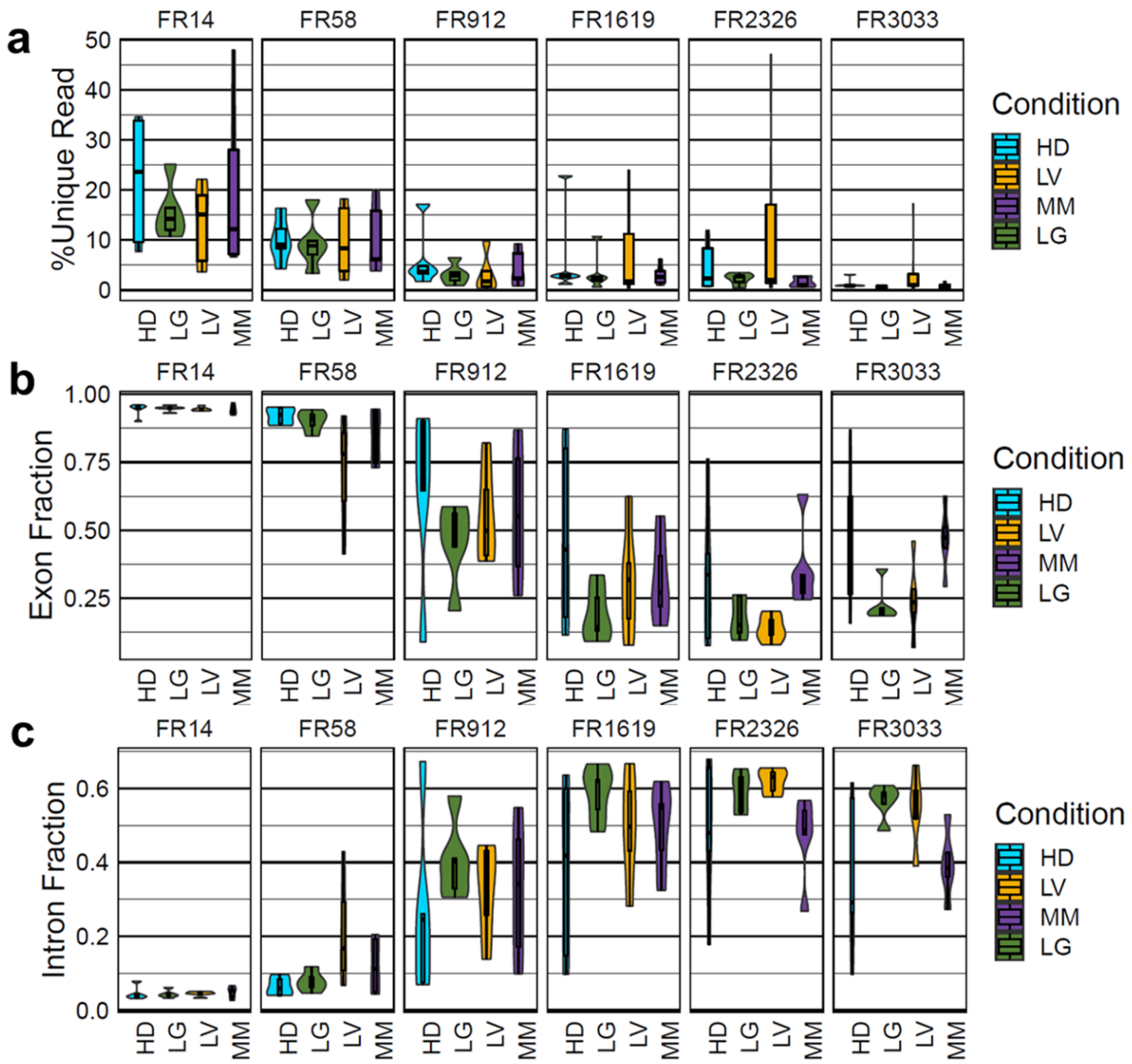

Supplementary Fig. 5. Distribution of % unique read, exon, and intron fraction. Violin plots across plasma fractions (FR14, FR58, FR912, FR1619, FR2326, and FR3033) grouped by each condition showing the respective fraction of **(a)** % unique read, **(b)** % reads mapping to exons (exon fraction), and **(c)** % read mapping to introns (intron fraction).

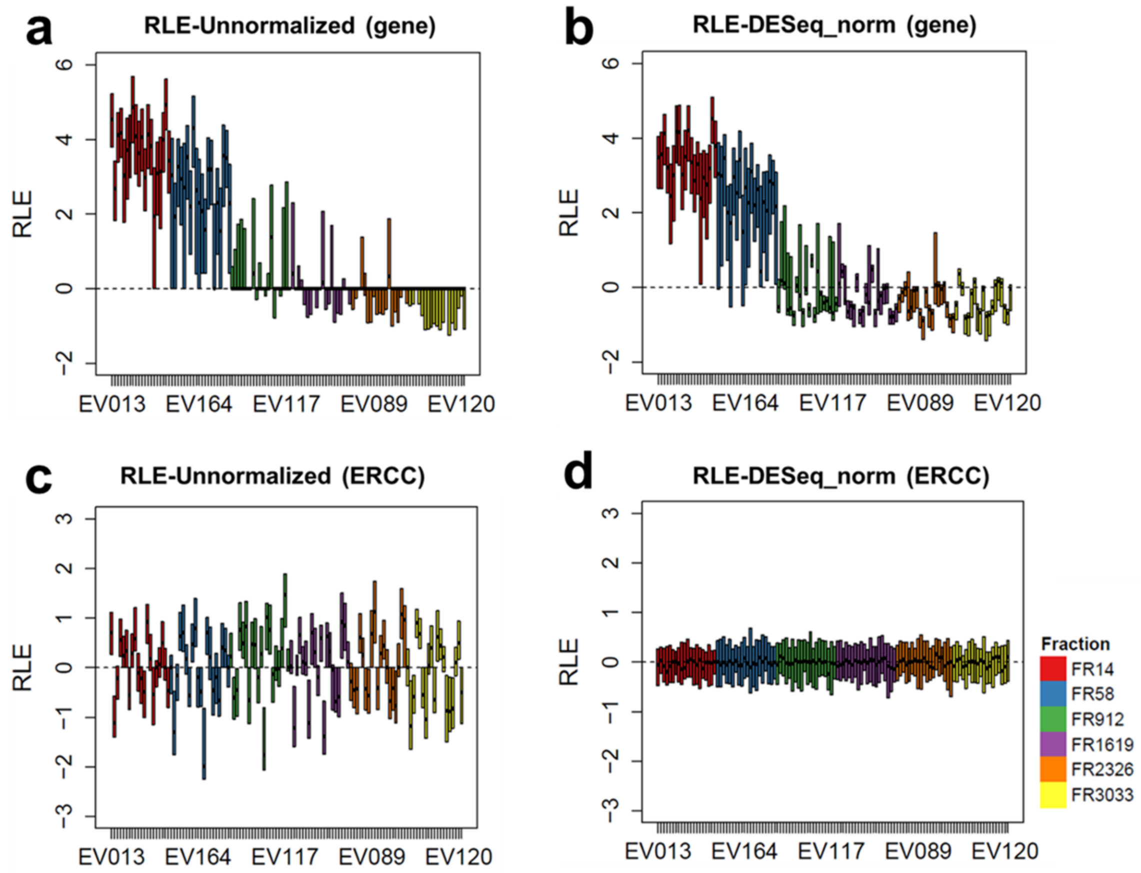

Supplementary Fig. 6. Comparison of unnormalized vs ERCC normalized counts. Box plot of relative log expression (RLE) across all sample set for **(a)** unnormalized and **(b)** ERCC normalized cf-mRNA counts. Box plot of relative log expression across all sample set for **(c)** unnormalized and **(d)** normalized ERCC counts. Relative log expression of FR14 (in red), FR58 (in blue), FR912 (in green), FR1619 (in purple), FR2326 (in orange), and FR3033 (in yellow) are shown.

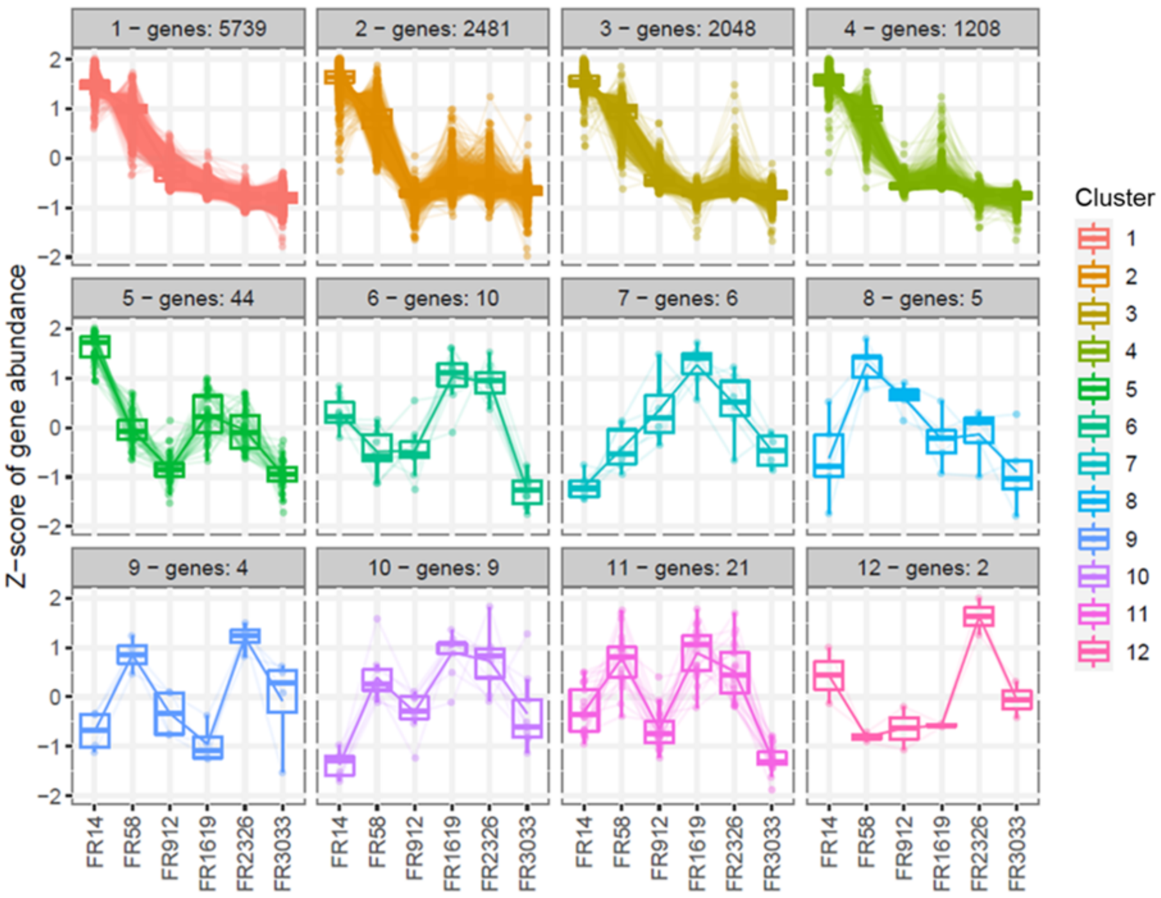

Supplementary Fig. 7. Result from degPatterns across plasma fractions. Box plot of z-score gene abundance of 12 distinct clusters across plasma fractions from degPatterns using differentially expressed genes (n = 11,577). Differentially expressed genes are derived from one-way ANOVA test with significance threshold of adjusted p value less than 0.05. Each box plot title includes cluster label and number of differentially expressed genes associated with the cluster.

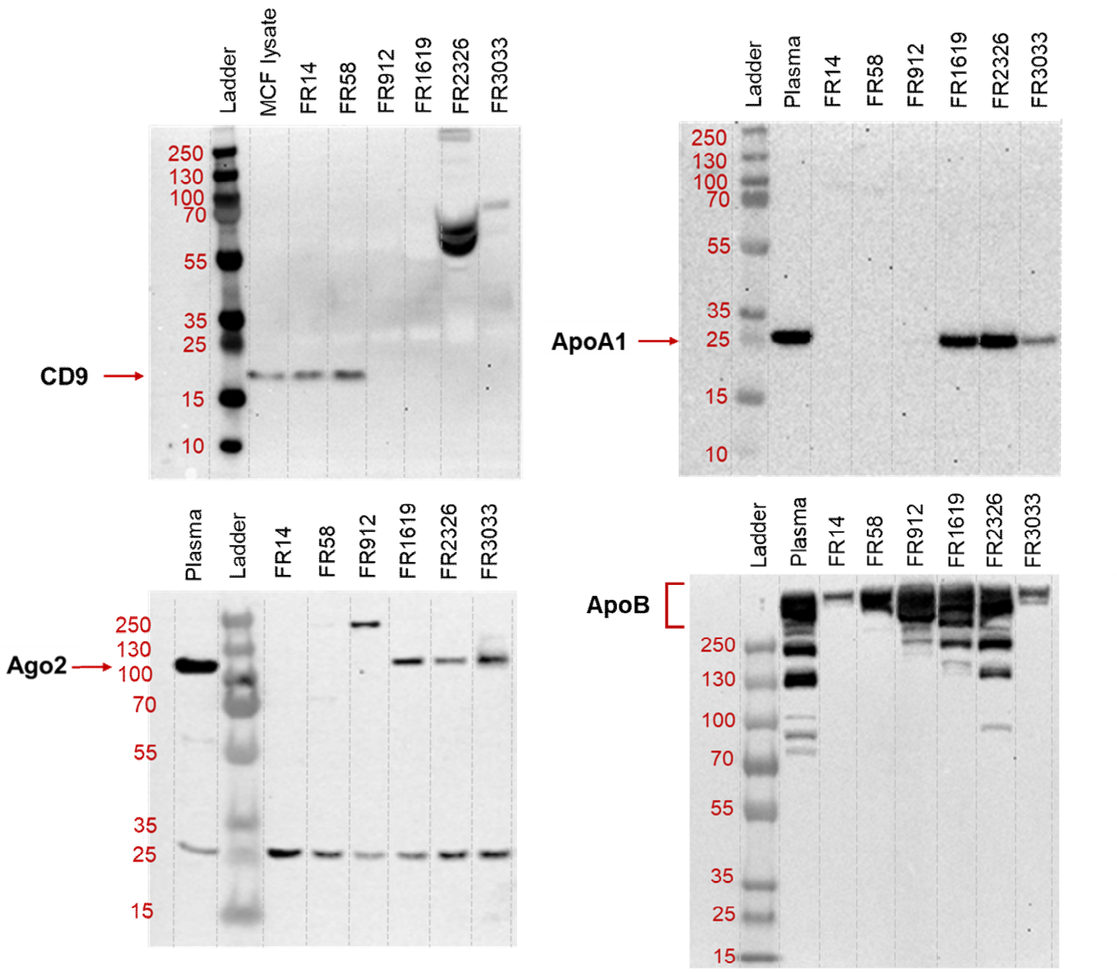

Supplementary Fig. 8. Result of immunoprecipitation across plasma fractions. Western blot analysis of anti-CD9, anti-ApoA1, anti-Ago2, anti-ApoB protein markers from plasma fractions using MCF lysate or plasma as positive controls.

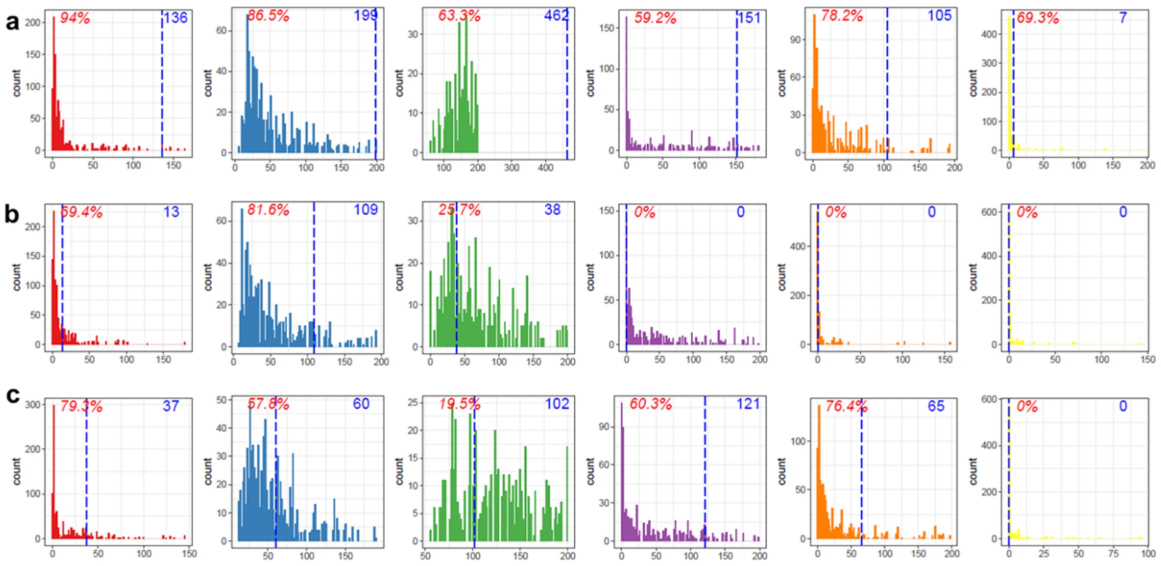

Supplementary Fig. 9. Permutation. Results of permutation by random sample shuffling with 1,000 rounds to test significance of the differential expression for each pairwise comparison between healthy and **(a)** lung cancer, **(b)** liver cancer, and **(c)** multiple myeloma within individual fractions. X-axis indicates number of significant genes (padj < 0.05 & log2FC <1) through random sample shuffling. Number in blue indicates number of significant genes with correct labeling (the blue line). Percentage in red indicates percentage of permutations with the number of DE genes below the blue line. The bar plots are organized by fractions from left to right: FR14 (in red), FR58 (in blue), FR912 (in green), FR1619 (in purple), FR2326 (in orange), and FR3033 (in yellow).

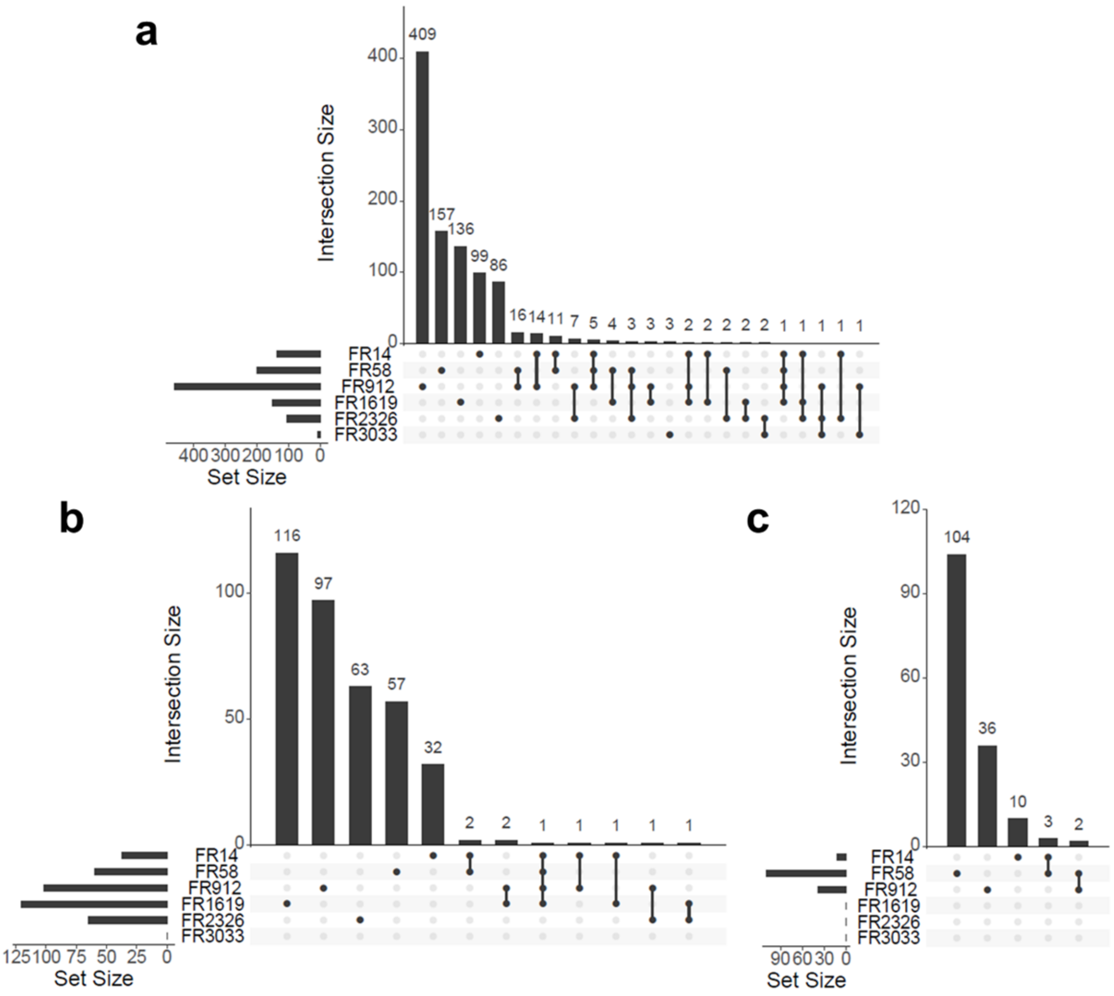

Supplementary Fig. 10. Number of differentially expressed genes. Upset plot of number of differentially expressed genes unique and shared across plasma between healthy and **(a)** lung cancer, **(b)** multiple myeloma, and **(c)** liver cancer. Set size represents number of differentially expressed genes across plasma fractions. Intersection size represents number of genes either unique or shared across plasma fractions (dots connected by lines).

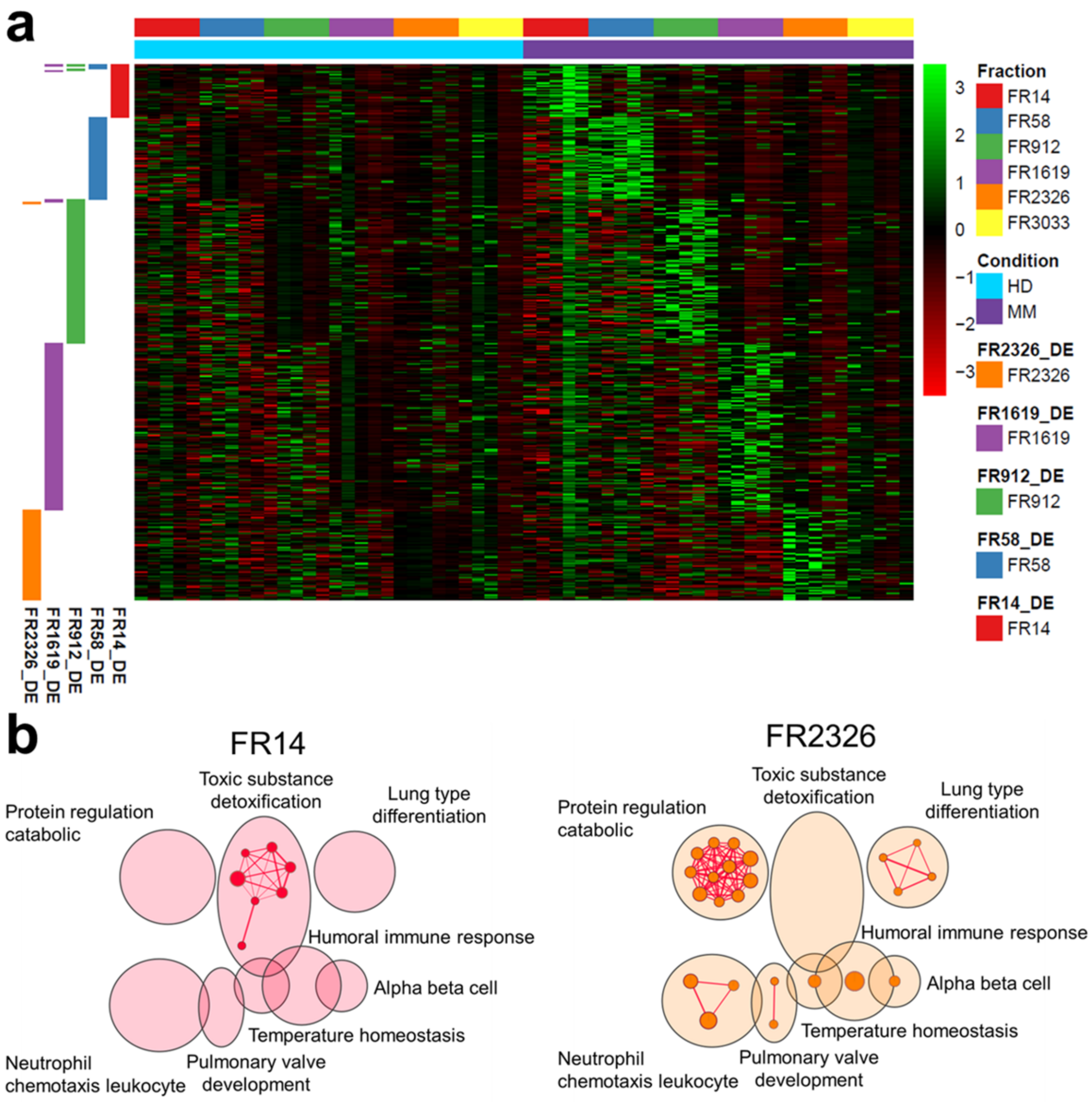

Supplementary Fig. 11. Selective Enrichment of Multiple Myeloma Differentiating cf-mRNA. (a) Heatmap of gene expression in multiple myeloma relative to healthy across fractions. **(b)** Enrichment map for multiple myeloma cancer DE genes found in fraction 14 (FR14) and fraction 2326 (FR2326) using Gene Ontology (Biological Properties) and Reactome. Cluster of nodes were automatically labeled using the AutoAnnotate from Cytoscape. Nodes were colored by fraction: FR14 (in red) and FR2326 (in orange).

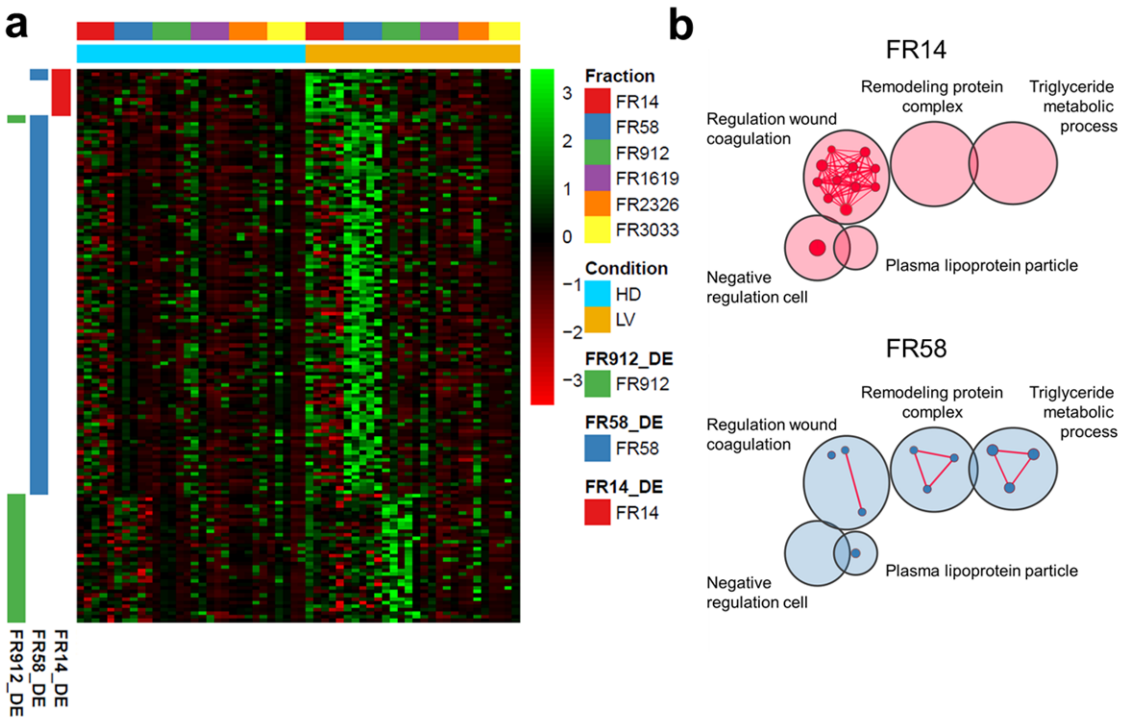

Supplementary Fig. 12. Selective Enrichment of Liver Cancer Differentiating cf-mRNA. (a) Heatmap of gene expression in liver cancer relative to healthy across fractions. **(b)** Enrichment map for liver cancer DE genes found in fraction 14 (FR14) and fraction 58 (FR58) using Gene Ontology (Biological Properties) and Reactome. Cluster of nodes were automatically labeled using the AutoAnnotate from Cytoscape. Nodes were colored by fraction: FR14 (in red) and FR58 (in blue).
