# Supplementary material for: Selective enrichment of plasma cell-free messenger RNA in cancer-associated extracellular vesicles
Source: Commun Biol. 2023 Aug 29;6:885. doi: 10.1038/s42003-023-05232-z (PMC10465482; doi:10.1038/s42003-023-05232-z)
Supplement: Supplementary file 3 — Description of Additional Supplementary Files [file 42003_2023_5232_MOESM3_ESM.pdf]

Description of Additional Supplementary Files

File name: Supplementary Data 1
Description: Raw particle counts for TEM imaging analysis

File name: Supplementary Data 2
Description: Clinical information
