# Supplementary material for: Selective enrichment of plasma cell-free messenger RNA in cancer-associated extracellular vesicles
Source: Commun Biol. 2023 Aug 29;6:885. doi: 10.1038/s42003-023-05232-z (PMC10465482; doi:10.1038/s42003-023-05232-z)
Supplement: Supplementary file 5 — Reporting Summary [file 42003_2023_5232_MOESM5_ESM.pdf]

nature portfolio

Corresponding author(s): Thuy Ngo

Last updated by author(s): Aug 17, 2023

Reporting Summary

Nature Portfolio wishes to improve the reproducibility of the work that we publish. This form provides structure for consistency and transparency in reporting. For further information on Nature Portfolio policies, see our Editorial Policies and the Editorial Policy Checklist.

Statistics

For all statistical analyses, confirm that the following items are present in the figure legend, table legend, main text, or Methods section.

n/a	Confirmed	
☐	☒	The exact sample size (*n*) for each experimental group/condition, given as a discrete number and unit of measurement
☐	☒	A statement on whether measurements were taken from distinct samples or whether the same sample was measured repeatedly
☐	☒	The statistical test(s) used AND whether they are one- or two-sided<br>*Only common tests should be described solely by name; describe more complex techniques in the Methods section.*
☐	☒	A description of all covariates tested
☐	☒	A description of any assumptions or corrections, such as tests of normality and adjustment for multiple comparisons
☐	☒	A full description of the statistical parameters including central tendency (e.g. means) or other basic estimates (e.g. regression coefficient) AND variation (e.g. standard deviation) or associated estimates of uncertainty (e.g. confidence intervals)
☐	☒	For null hypothesis testing, the test statistic (e.g. *F*, *t*, *r*) with confidence intervals, effect sizes, degrees of freedom and *P* value noted<br>*Give P values as exact values whenever suitable.*
☒	☐	For Bayesian analysis, information on the choice of priors and Markov chain Monte Carlo settings
☐	☒	For hierarchical and complex designs, identification of the appropriate level for tests and full reporting of outcomes
☒	☐	Estimates of effect sizes (e.g. Cohen's *d*, Pearson's *r*), indicating how they were calculated

Our web collection on statistics for biologists contains articles on many of the points above.

Software and code

Policy information about availability of computer code

Data collection	*Provide a description of all commercial, open source and custom code used to collect the data in this study, specifying the version used OR state that no software was used.*

Data analysis

The quality of the RNA sequencing reads were checked using FastQC (v0.11.8) [50, 51] and RSeQC (v3.0.0) [52]. Reads were aligned to the human genome assembly (hg38, ensembl annotation; v94) and ERCC RNA spike-in sequences using the STAR aligner (v2.5.3a) [53] with two pass mode flag. Following adapter trimming and alignment using STAR (ver 2.5.3), bigwig coverage tracks were generated from each sample alignment file using bedtools (ver 2.27.1). Sample bigwig files were then visualized using the ggcoverage R package (ver 0.7.1) over the genomic range of the housekeeping gene ACTB as well as ALB. Read counts for each gene were calculated using the htseq-count tool (v0.11.2) [54] in intersection-strict mode.

For each sample, we calculated exon, intron, and protein coding fractions (CDS exons) using RSeQC (v3.0.0) [52]. The unnormalized protein coding transcripts were then normalized using ERCC RNA spike-in control as size factors in DESeq2 (v1.22.2) [55]. The relative log expression was obtained from RUVseq package (v1.16.1) [44]. Differentially expressed genes between case/control of fractionated samples were identified using adjusted p-value (padj) < 0.05 and log2 fold change (log2FC) >1 from DESeq2 (v1.22.2). For DegPattern analysis, we employed R package DEGreport (v1.18.1).

Pathway enrichment analysis was performed according to Reimand et al. [56]. We created generic enrichment map (GEM) files for pathway analysis using gprofiler, which performs functional profiling of gene list from large-scale experiment (https://biit.cs.ut.ee/gprofiler/) [57]. For visualization and network enrichment analysis, GEM files were imported to Cytoscape (v3.9.0) (http://www.cytoscape.org/), and GMT file from the g:Profiler website containing data source was specified. EnrichmentMap in Cytoscape (http://www.baderlab.org/Software/EnrichmentMap) was used to build the network [56].

For manuscripts utilizing custom algorithms or software that are central to the research but not yet described in published literature, software must be made available to editors and reviewers. We strongly encourage code deposition in a community repository (e.g. GitHub). See the Nature Portfolio guidelines for submitting code & software for further information.

Data

Policy information about availability of data

All manuscripts must include a data availability statement. This statement should provide the following information, where applicable:

- Accession codes, unique identifiers, or web links for publicly available datasets
- A description of any restrictions on data availability
- For clinical datasets or third party data, please ensure that the statement adheres to our policy

Sequencing data (GSE205301) is deposited in the Gene Expression Omnibus Repository. In-house scripts and all numerical source data used in this manuscript for graphs and analyses, which include data processing, downstream analysis, and the scripts used to generate figures are publicly available on Github repository: https://github.com/pyunjis/EV-RNA.

Research involving human participants, their data, or biological material

Policy information about studies with human participants or human data. See also policy information about sex, gender (identity/presentation), and sexual orientation and race, ethnicity and racism.

Reporting on sex and gender

All samples were collected under OHSU institutional review board (IRB) approved protocols. All donors gave written informed consents for research use. Gender was determined based on self-reporting.

Reporting on race, ethnicity, or other socially relevant groupings

Socially relevant categorization variable was not used.

Population characteristics

Samples for analysis were matched between cancer and control groups with respect to age and gender of participants. The clinical information regarding study participants are given in the Supplementary Table S2.

Recruitment

Blood samples from control individuals and patients with multiple myeloma, liver cancer, and lung cancer were obtained from Oregon Health and Science University (OHSU) by Knight Cancer Institute Biolibrary and Oregon Clinical and Translational Research Institute (OCTRI).

Ethics oversight

All samples were collected under OHSU institutional review board (IRB) approved protocols.

Note that full information on the approval of the study protocol must also be provided in the manuscript.

Field-specific reporting

Please select the one below that is the best fit for your research. If you are not sure, read the appropriate sections before making your selection.

☒ Life sciences ☐ Behavioural & social sciences ☐ Ecological, evolutionary & environmental sciences

For a reference copy of the document with all sections, see nature.com/documents/nr-reporting-summary-flat.pdf

Life sciences study design

All studies must disclose on these points even when the disclosure is negative.

Sample size	The sample set includes 5 individuals for each cancer type and control group: multiple myeloma, liver cancer, lung cancer, and control groups with 6 plasma size fractionated samples (FR14, FR58, FR912, FR1619, FR2326, and FR3033 corresponding to large particles, medium particles, small particles, early-eluting, middle-eluting, and late-eluting protein fractions) respectively. These result in total of 120 size-fractionated samples for RNA sequencing.
Data exclusions	We retained total cell-free RNA counts with more than five reads in at least one plasma fraction from all samples. The total cell-free RNA counts were then filtered by protein-coding biotype using human genome assembly (hg38, ensembl annotation; v94), resulting in identification of 11,609 expressed cell-free mRNA transcripts. The relative log expression was obtained from RUVseq package (v1.16.1) [44]. 2 out of 120 of the plasma fractions were excluded due to the being an outlier through clustering of genes by expression pattern.
Replication	Each sample was sequenced once without replicates.
Randomization	All fractionated plasma samples isolated by SEC were randomized to reduce sample batch effects. The purified RNA samples were randomly assigned to prepare the RNA-seq library and were randomly distributed over three NovaSeq S4 lanes to reduce the batch effects. To control processing and normalization, a consistent amount of ERCC RNA control mix was spiked into plasma fractions.
Blinding	Investigators were not blinded during sample collection analysis due to the collection process from specific clinics.

Reporting for specific materials, systems and methods

We require information from authors about some types of materials, experimental systems and methods used in many studies. Here, indicate whether each material, system or method listed is relevant to your study. If you are not sure if a list item applies to your research, read the appropriate section before selecting a response.

Materials & experimental systems

n/a	Involved in the study
☐	☒ Antibodies
☐	☒ Eukaryotic cell lines
☒	☐ Palaeontology and archaeology
☒	☐ Animals and other organisms
☒	☐ Clinical data
☒	☐ Dual use research of concern
☒	☐ Plants

Methods

n/a	Involved in the study
☒	☐ ChIP-seq
☒	☐ Flow cytometry
☒	☐ MRI-based neuroimaging

Antibodies

Antibodies used	For immunoprecipitation, mouse monoclonal anti-Ago2 (Abcam, ab57113), anti-CD9 antibody (Abcam, ab58989), anti-Apolipoprotein A1 (Santa Cruz Biotechnology, sc-376818), ApoB antibody from Santa Cruz (sc-13538) or mouse normal IgG antibodies (Santa Cruz Biotechnology, sc-2025) were used to incubate with the magnetic bead slurry. The anti-Argonaut-2 antibody (Abcam, ab32381), anti-CD9 (Abcam, ab223052), and anti-apolipoprotein A1 (Abcam, ab64308) and goat polyclonal anti-ApoB100 antibody (R&D Systems, AF3260) were used as primary antibodies. After washing with 1X TBST, the membrane was incubated with horseradish peroxidase conjugated anti-rabbit secondary antibodies (Cell Signaling, 7074) or anti-goat secondary antibodies (Promega, V8051) respectively.
Validation	Validations with positive and negative control were shown by each company's websites.

Eukaryotic cell lines

Policy information about cell lines and Sex and Gender in Research

Cell line source(s)	MCF7 breast cancer cell line from ATCC was used to collect MCF lysate for western blot control.
Authentication	MCF7 cell line was authenticated from ATCC with certicate of analysis.
Mycoplasma contamination	Cell line was negative for mycoplasma contamination.
Commonly misidentified lines (See ICLAC register)	*Name any commonly misidentified cell lines used in the study and provide a rationale for their use.*
